# Attitudes and preferences for the clinical management of hypertension and hypertension-related cerebrovascular disease in the general practice: results of the Italian hypertension and brain survey

**DOI:** 10.1186/s40885-017-0066-0

**Published:** 2017-05-15

**Authors:** Giuliano Tocci, Arrigo F. Cicero, Massimo Salvetti, Maria Beatrice Musumeci, Andrea Ferrucci, Claudio Borghi, Massimo Volpe

**Affiliations:** 1 Hypertension Unit, Division of Cardiology, Department of Clinical and Molecular Medicine, Faculty of Medicine and Psychology, University of Rome Sapienza, Sant’Andrea Hospital, Via di Grottarossa 1035, Rome, 00189 Italy; 2 Division of Internal Medicine, University of Bologna, Bologna, Italy; 3 Department of Clinical and Experimental Sciences, University of Brescia, Brescia, Italy; 4 IRCCS Neuromed, Pozzilli (IS), Italy

**Keywords:** Hypertension management, Hypertension control, Cerebrovascular disease, Stroke, Clinical survey

## Abstract

**Background:**

The aim of this survey was to evaluate attitudes and preferences for the clinical management of hypertension and hypertension-related cerebrovascular diseases (CVD) in Italy.

**Methods:**

A predefined 16-item survey questionnaire was anonymously administered to a large community sample of general practitioners (GPs), trained by specialized physicians (SPs), who have been included in an educational program between January and November 2015.

**Results:**

A total of 591 physicians, among whom 48 (8%) training SPs and 543 (92%) trained GPs, provided 12,258 valid answers to the survey questionnaire. Left ventricular hypertrophy was considered the most frequent marker of hypertension-related organ damage, whereas atrial fibrillation and carotid atherosclerosis were considered relatively not frequent (10–20%). The most appropriate blood pressure (BP) targets to be achieved in hypertensive patients with CVD were <140/90 mmHg for SPs and <135/85 mmHg for GPs. To achieve these goals, ACE inhibitors were considered the most effective strategies by GPs, whereas SPs expressed a preference for ARBs, both in monotherapies and in combination therapies with beta-blockers.

**Conclusions:**

This survey demonstrates that Italian physicians considered left ventricular hypertrophy frequently associated to CVD and that drugs inhibiting the renin-angiotensin system the most appropriate therapy to manage hypertension and hypertension-related CVD.

**Electronic supplementary material:**

The online version of this article (doi:10.1186/s40885-017-0066-0) contains supplementary material, which is available to authorized users.

## Background

Effective control of high blood pressure (BP) reduces incidence of major cardiovascular complications, and improves event-free survival from cerebrovascular diseases (CVD), mostly stroke [1]. Despite these beneficial effects, observed BP control rates are relatively poor, worldwide [2, 3].

In order to improve BP control, several interventions have been proposed and applied in various countries, including Italy. Among these, a closer attention to global cardiovascular risk stratification and a proper selection of antihypertensive drug therapies on the basis of individual global CV risk profile have emerged as the best way to ameliorate hypertension management and control [4–6]. However, despite the large availability of practical recommendations and guidelines as well as of different and well-tolerated antihypertensive drug classes, some aspects of the clinical management of hypertension still represent a difficult clinical task. For example, presence of comorbidities, such as CVD, may affect both therapeutic choices among different antihypertensive drugs, as well as BP goals. This was at least, in part, due to the fact that recent randomized, controlled clinical trials, performed in patients at high or very high CV risk, have often provided conflicting results [7–11]. For these reasons, definite evidence supporting the use of specific drug classes or molecules, as well as the application of diagnostic tests or BP targets in these very high-risk hypertensive patients are relatively lacking. Even the most recent set of hypertension European guidelines has acknowledged this aspect, and discussed on how and how much BP levels should be reduced in hypertensive patients with comorbidities [12].

More recently, an extensive use of epidemiological surveys and observational studies has emerged as a valuable option to evaluate physicians’ workflow, particularly when managing hypertensive outpatients at different CV risk [13–15]. In this view, we had the possibility to analyse survey questionnaires, which evaluated physicians’ diagnostic and therapeutic positions when managing patients with hypertension and high CV risk [16, 17].

In the present survey, we evaluated the clinical attitudes and preferences for the management of patients with hypertension and hypertension-related CVD, expressed by a large sample of physicians in Italy.

## Methods

### Aims of the survey

The primary aim of this survey was to evaluate the clinical attitudes and preferences of both general practitioners (GPs) and specialized physicians (SPs), who were included in an educational program performed in Italy in 2015.

Secondary aims of the survey were to analyse pharmacological preferences (monotherapy vs. combination therapy, and type of combination therapies) in patients with hypertension and CVD.

### Methodology of the survey

The methodology of the study has been previously described [16]. Briefly, this is an observational, non-interventional, cross-sectional study, designed to evaluate physicians’ attitudes and preferences for the daily clinical management of hypertension through the administration of a specifically designed survey questionnaire. This survey generated from an educational training program, devoted to clinicians involved in the clinical management of patients with hypertension and comorbidities in Italy [16]. This educational program was originally planned to cover four main areas of hypertension-related comorbidities (including heart, lung, brain and kidney) during a 4-year period (one area for each year) [16].

The study conformed to the Declaration of Helsinki and its subsequent modifications. Confidentiality on demographic and personal data of each physician included in the present survey was carefully preserved and strictly protected during each phase of the study. No access was made to individual data of neither physicians’ own patients nor their medical databases. Written consent to participate to the educational program was obtained by all involved physicians.

### Survey questionnaire

The survey questionnaire included a total of 16 questions addressing the following items: 1) estimated concomitant prevalence of hypertension and CVD and prevalence of hypertension-related markers of organ damage and comorbidities in patients with hypertension and CVD (questions num. 01–06); 2) diagnostic options to assess the presence of CVD in hypertensive patients (questions num. 07–08); 3) BP targets and the most appropriate therapeutic targets to be achieved in a setting of clinical practice, when managing hypertensive patients with CVD (question num. 09–10 and 13–14); 4) preferences for antihypertensive drug classes in hypertensive patients with CVD to be used as first line therapy (monotherapy) or combination therapy (questions num. 11–12 and 15–16). The full survey questionnaire is reported in Additional file 1: Table S1 (online available).

### Physicians’ engagement

Physicians’ engagement was carried out during the first 6 months of 2015. As per study protocol [16], participants involved in this educational program were randomly designated from a community sample of physicians, operating in different clinical settings (outpatients clinics and/or in-hospital divisions), geographical locations (north-east, north-west, center and south of Italy), and age of clinical activity, in order to have a representative sample of physicians who have practice of patients with hypertension and hypertension-related comorbidities in Italy. SPs may include doctors with at least one of the following professional category: cardiology, neurology, geriatric, nephrology and internal medicine.

Physicians were invited to participate to an educational program, aimed at improving knowledge on hypertension-related cardiovascular diseases and implementing strategies for achieving better BP control in their practice. Educational program was structured into two distinct sections: one start-up meeting, held in January 2015 and pointed to SPs, and local meetings, distributed throughout the whole Italian territory, during which SPs involved in the start-up meeting trained GPs. Before starting each educational meeting, involved physicians were asked to fill the survey questionnaire anonymously. The entire survey questionnaire was completed by participants on-site and then delivered to the data collection centre. Physicians who completed the survey did not receive any compensation for their participation.

### Statistical analysis

All data derived from the survey questionnaires were reported into a computerised sheet (Microsoft Excel, Microsoft Office™). Then, analyses were made to generate proportions of individual answers to each question of the survey questionnaire. A separate sheet, containing graphs, was produced for the 16 questions of the survey questionnaire. Data were presented as a percentage of the total answers to each question.

## Results

### Population sample and questionnaire

Overall, 591 physicians (342 males, mean age 58.2 ± 6.4 years, average age of medical activity 26.5 ± 7.3 years) were included in the survey. Among these, 48 (8.1%) were training SPs and 543 (91.9%) were trained GPs. Involved physicians provided 12,599 answers to the survey questionnaire, among which 312 (2.5%) were considered inappropriate or incorrect and 29 (0.2%) were missing or not reported. Thus, a total of 12,258 valid answers were included in the present analysis, which represents the 97.3% of the overall results generated by the survey questionnaire.

### Analysis of the survey questionnaire

#### Estimated prevalence of organ damage and comorbidities in hypertension

As shown in Table 1, the vast majority of physicians, mostly GPs (81.2%), identified left ventricular hypertrophy as the most frequent marker of organ damage compared to carotid atherosclerosis (7.9%), microalbuminuria or proteinuria (5.9%), impaired renal (4.5%) or vascular (0.9%) function. In particular, about half of involved physicians reported that this marker of cardiac organ damage can be found in approximately 21–40% of their hypertensive outpatients, whereas about one third of physicians reported higher estimated prevalence, without relevant differences between the two groups.

**Table 1:** Perceived prevalence of markers of hypertension-related organ damage and cerebrovbascular diseases, including transient ischemic attack and stroke, according to physicians’ answers to survey questionnaire [questions num. 01–06]

Question (num/text)	Answers (%)		
	Overall (*N* = 591)	SPs (*n* = 48)	GPs (*n* = 543)
Q01. Which is the most prevalent marker of organ damage do you find in patients with hypertension in your clinical practice?			
Left Ventricular Hypertrophy	469 (80.9)	37 (77.1)	432 (81.2)
Carotid Atherosclerosis	46 (7.9)	6 (12.5)	40 (7.5)
Microalbuminuria or Proteinuria	34 (5.9)	5 (10.4)	29 (5.5)
Impaired eGFR or CrCl	26 (4.5)	0 (0.0)	26 (4.9)
Impaired ABI or PWV	5 (0.9)	0 (0.0)	5 (0.9)
Q02. Which is the prevalence of cardiac organ damage (i.e. left ventricular hypertrophy) do you find in patients with hypertension in your clinical practice?			
10–20%	110 (18.9)	7 (14.6)	103 (19.3)
21–40%	278 (47.8)	24 (50.0)	254 (47.6)
41–50%	120 (20.6)	10 (20.8)	110 (20.6)
> 50%	74 (12.7)	7 (14.6)	67 (12.5)
Q03. Which is the prevalence of renal organ damage (i.e. MAU, proteinuria, reduced eGFR or creatinine clearance) do you find in patients with hypertension in your clinical practice?			
10–20%	196 (33.7)	17 (35.4)	179 (33.6)
21–40%	267 (46.0)	26 (54.2)	241 (45.2)
41–50%	88 (15.1)	4 (8.3)	84 (15.8)
> 50%	30 (5.2)	1 (2.1)	29 (5.4)
Q04. Which is the prevalence of vascular organ damage (i.e. carotid or peripheral atherosclerosis) do you find in patients with hypertension in your clinical practice?			
10–20%	430 (74.3)	33 (68.8)	397 (74.8)
21–40%	132 (22.8)	15 (31.3)	117 (22.0)
41–50%	11 (1.9)	0 (0.0)	11 (2.1)
> 50%	6 (1.0)	0 (0.0)	6 (1.1)
Q05. Which is the prevalence of cerebrovascular disease (i.e. transient ischemic attack) do you find in patients with hypertension in your clinical practice?			
10–20%	388 (67.6)	37 (82.2)	351 (66.4)
21–40%	143 (24.9)	8 (17.8)	135 (25.5)
41–50%	35 (6.1)	0 (0.0)	35 (6.6)
> 50%	8 (1.4)	0 (0.0)	8 (1.5)
Q06. Which is the prevalence of cerebrovascular disease (i.e. stroke) do you find in patients with hypertension in your clinical practice?			
10–20%	432 (75.0)	42 (93.3)	390 (73.4)
21–40%	116 (20.1)	3 (6.7)	113 (21.3)
41–50%	24 (4.2)	0 (0.0)	24 (4.5)
> 50%	4 (0.7)	0 (0.0)	4 (0.8)

*SPs* specialized physicians, *GPs* general practitioners, *MAU* microalbuminuria, *eGFR* estimated glomerular filtration rate

Renal organ damage was considered to be frequent by 54% of SPs and 45% of GPs, although about one third of both groups of physicians considered this marker relatively not frequent in their clinical practice. At the same time, vascular organ damage (either carotid or peripheral atherosclerosis) was considered relatively not frequent in hypertensive outpatients by the vast majority of involved physicians. Of note, estimated prevalence of CVD, including transient ischemic attack and stroke, was reported to be relatively low mostly in those hypertensive outpatients followed by SPs compared to those followed by GPs.

#### Preferred diagnostic options

As shown in Table 2, about half of GPs considered the echocardiogram as the most appropriate diagnostic tool to be used in patients with hypertension and history of CVD, followed by carotid vascular ultrasound, whereas this latter was the preferred option by similar proportion of SPs compared the former one.

**Table 2:** Preferred diagnostic tools used in the clinical practice in patients with hypertension either to assess [question num. 07] or to exclude presence of CVD [question num. 08], including transient ischemic attack and stroke, according to physicians’ answers to survey questionnaire

Question (num/text)	Answers (%)		
	Overall (*N* = 591)	SPs (*n* = 48)	GPs (*n* = 543)
Q07. Which diagnostic tool do you think is the most appropriate in patients with hypertension and CVD (i.e. transient ischemic attack or stroke) in your clinical practice?			
Echocardiogram	294 (50.8)	10 (20.8)	284 (53.5)
Carotid Vascular Ultrasound	230 (39.7)	25 (52.1)	205 (38.6)
Transcranic Vascular Ultrasound	14 (2.4)	1 (2.1)	13 (2.4)
24-h ABPM	39 (6.7)	11 (22.9)	28 (5.3)
Central Aortic Pressure and/or PWV	2 (0.3)	1 (2.1)	1 (0.2)
Q08. Which diagnostic tool do you think is the most appropriate in patients with hypertension to exclude the presence of CVD (i.e. transient ischemic attack or stroke) in your clinical practice?			
Carotid Vascular Ultrasound	331 (57. 3)	14 (29.2)	317 (59.8)
Transcranic Vascular Ultrasound	26 (4.5)	0 (0.0)	26 (4.9)
Electroencefalogram	7 (1.2)	0 (0.0)	7 (1.3)
Brain Imaging (CT or MR)	179 (31.0)	30 (62.5)	149 (28.1)
Angio-MR	35 (6.1)	4 (8.3)	31 (5.8)

*SPs* specialized physicians, *GPs* general practitioners, *ABPM* ambulatory blood pressure monitoring, *PWV* pulse wave velocity, *CT* computer tomography, *MR* magnetic resonance

On the other hand, the majority of GPs considered the carotid vascular ultrasound the most appropriate diagnostic tool to be used in patients with hypertension for excluding the presence of CVD in their clinical practice, whereas, the majority of SPs expressed a clear preference for brain imaging techniques, including CT or MR.

#### Preferred therapeutic targets and BP goals

As shown in Table 3, in hypertensive patients with TIA, the achievement of the recommended BP targets represents the key priority according to about half of GPs (45.3%), followed by absolute BP reductions (36.4%) and protection from hypertension-related organ damage (13.0%). Conversely, SPs equally identified protection from organ damage (42.6%) and achievement of the recommended BP targets (40.4%) as the most important therapeutic targets, followed by absolute reductions of BP levels. Of note, minor proportions of both groups of physicians considered an improved adherence to prescribed medications of clinical relevance, while reduction of drug-related side effects and adverse reactions was only marginally considered by both groups of physicians.

**Table 3:** Preferred therapeutic targets to be achieved under pharmacological therapy in hypertensive patients with transient ischemic attack [question num. 10] and in those with stroke [question num. 14]

Question (num/text)	Answers (%)		
	Overall (*N* = 591)	SPs (*n* = 48)	GPs (*n* = 543)
Q10. Which is the most important target do you wish to achieve in patients with hypertension an transient ischemic attack in your clinical practice?			
Reduce BP levels	198 (34.3)	5 (10.6)	193 (36.4)
Achieve the recommended BP targets	259 (44.9)	19 (40.4)	240 (45.3)
Protect from organ damage	89 (15.4)	20 (42.6)	69 (13.0)
Improve adherence and persistence on therapy	30 (5.2)	3 (6.4)	27 (5.1)
Reduce side effects and adverse reactions	1 (0.2)	0 (0.0)	1 (0.2)
Q14. Which is the most important target do you wish to achieve in patients with hypertension and previous stroke in your clinical practice?			
Reduce BP levels	203 (36.1)	5 (10.6)	198 (38.4)
Achieve the recommended BP targets	260 (46.3)	18 (38.3)	242 (47.0)
Protect from organ damage	75 (13.3)	19 (40.4)	56 (10.9)
Improve adherence and persistence on therapy	23 (4.1)	5 (10.6)	18 (3.5)
Reduce side effects and adverse reactions	1 (0.2)	0 (0.0)	1 (0.2)

*SPs* specialized physicians, *GPs* general practitioners, *BP* blood pressure

Also in the clinical management of hypertensive outpatients with stroke, the achievement of the recommended BP targets was considered the most important therapeutic target by 47% of GPs, followed by absolute BP reductions (38.4%) and protection from hypertension-related organ damage (10.9%), whereas SPs gave similar clinical relevance to protection from organ damage (40.4%) and achievement of the recommended BP targets (38.3%). Also in this case, adherence to prescribed medications was relatively partially considered by SPs (10.6%) and GPs (3.5%). Of note, reduction of drug-related side effects and adverse reactions was basically not considered of clinical relevance by both groups of physicians.

**Fig. 1:**
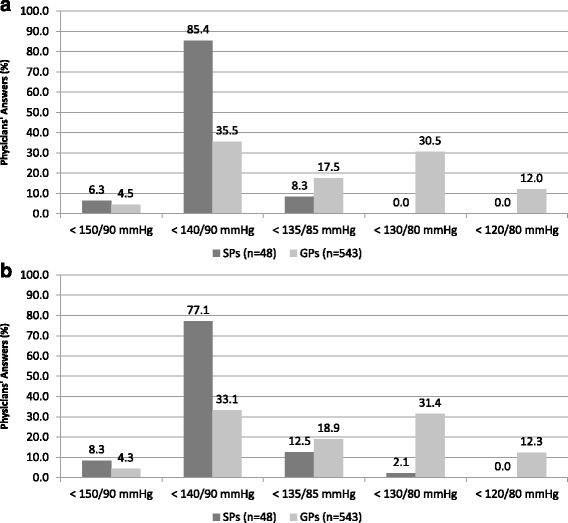
Blood pressure targets considered appropriate in hypertensive patients with transient ischemic attack [question num. 09] (panel **a**) and in those with stroke [question num. 13] (panel **b**) according to physicians’ answers to survey questionnaire. In the figure: SPs, specialized physicians; GPs, general practitioners

Differences between two groups of physicians were observed with regard to BP goals in hypertensive outpatients with TIA (Fig. 1a). Indeed, the vast majority of SPs considered 140/90 mmHg as optimal BP targets, whereas about one third of GPs identified the same BP goals. About one third of GPs (30.5%) also considered 130/80 mmHg, whereas minor proportions identified 135/85 mmHg or 120/80 mmHg as appropriate BP targets to be achieved in hypertensive outpatients with TIA. Similar distribution of preferences was also observed with regard to BP goals in hypertensive outpatients with stroke (Fig. 1b). The majority of SPs clearly identified 140/90 mmHg as the most appropriate BP goals in these very high-risk hypertensive outpatients, whereas only 33.1% of GPs expressed the same preference. About one third of GPs (31.4%) considered 130/80 mmHg, whereas minor proportions identified 135/85 mmHg or 120/80 mmHg as appropriate BP goals in hypertensive outpatients with stroke.

#### Preferred options for pharmacological therapies

**Fig. 2:**
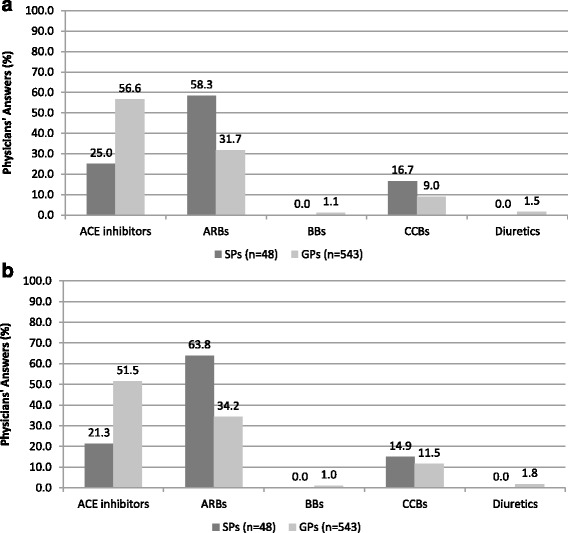
Antihypertensive drug strategy considered appropriate as firs line therapy in hypertensive patients with transient ischemic attack [question num. 11] (panel **a**) and in those with stroke [question num. 15] (panel **b**) according to physicians’ answers to survey questionnaire. In the figure: SPs, specialized physicians; GPs, general practitioners; ACE, angiotensin converting enzyme; ARBs, angiotensin receptor blockers; BBs, beta-blockers; CCBs, calcium–channel blockers

In hypertensive outpatients with TIA (Fig. 2a), angiotensin-converting enzyme (ACE) inhibitors was considered the preferred first-line option by about 57% of GPs, whereas 58% of SPs clearly identified angiotensin receptor blockers (ARBs) as first line therapy. Similarly, about one third of SPs GPs expressed a preference for either ACE inhibitors or ARBs, respectively, whereas only a minority of both groups of physicians took into consideration other antihypertensive drug classes, mostly calcium-channel blockers as first line therapy.

In hypertensive outpatients with stroke (Fig. 2b), about 64% of SPs expressed a preference for ARB-based monotherapy, and only 21% for ACE-inhibitor-based monotherapy. Conversely, about 51% of GPs preferred an ACE-inhibitor-based monotherapy, and 34% of GPs for ARB-based monotherapy. Even in this case, relatively low proportions of both groups of physicians reported to have a preference for other drugs in monotherapy, mostly including calcium-channel blockers.

**Fig. 3:**
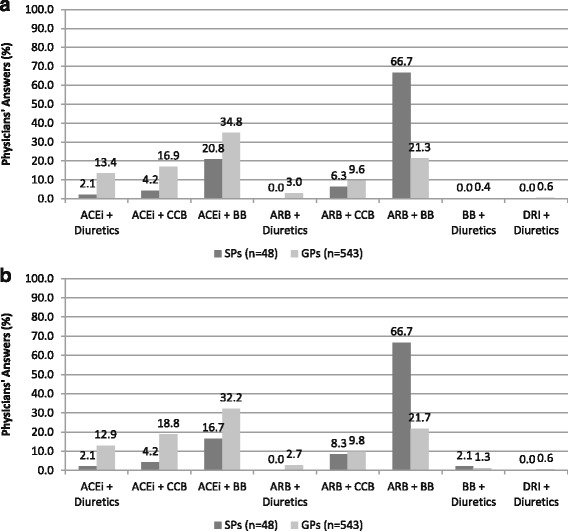
Antihypertensive drug strategy considered appropriate as combination therapy in hypertensive patients with transient ischemic attack [question num. 12] (panel **a**) and in those with stroke [question num. 16] (panel **b**) according to physicians’ answers to survey questionnaire. In the figure: SPs, specialized physicians; GPs, general practitioners; ACE, angiotensin converting enzyme; ARBs, angiotensin receptor blockers; BBs, beta-blockers; CCBs, calcium-channel blockers; DRI, direct renin inhibitors

Combination therapies based on ACE inhibitors with beta-blockers, diuretics or calcium-channel blockers represented the preferred options for treating patients with hypertension and TIA by GPs (Fig. 3a). On the contrary, the majority of SPs expressed a clear preference for combination therapies based on ARBs and beta-blockers (66.7%), whereas minor proportions reported to use combination therapies based on ARBs plus calcium-channel blockers.

Similar proportions were observed for physicians’ preferences with regard to different combination therapies used for treating hypertensive patients with stroke (Fig. 3b). In particular, combination therapies based on ACE inhibitors plus beta-blockers, diuretics or calcium channel blockers were preferred by GPs compared to SPs who reported a predominant use of combination therapies based on ARBs and beta-blockers (66.7%).

## Discussion

It is well known that hypertension management and control have been not achieved for many years, and that this relative failure has largely contributed to a persistently high burden of hypertension-related CVD, mostly including TIA and stroke, worldwide. It has been also shown that many factors can be advocated to try to explain the reported poor rates of BP control observed in various Western Countries, including Italy. Among these factors, patients’ clinical characteristics and behaviours (i.e. very high individual global cardiovascular risk profile, low adherence to prescribed medications, high rates of drug discontinuations), as well as poor effectiveness of antihypertensive drug strategies (i.e. persistently high use of monotherapies, inappropriate dosages, either not recommended or extremely complex combination therapies) have been acknowledged.

As a matter of fact, all these items are mostly focused on hypertensive outpatients rather than on treating physicians. Indeed, minor data are available to evaluate physicians’ preferences and behaviours in the clinical management of hypertension in real practice. In this view, we recently analysed the preferred options for the clinical management of outpatients with hypertension and hypertension-related diseases expressed by Italian physicians with different medical skills [16–19]. These studies highlighted some relevant discrepancies between recommendations from international guidelines and procedures applied in the clinical practice [16–19].

First of all, concomitant presence of hypertension and CVD was considered to be relatively not frequent in a setting of clinical practice. The vast majority of both groups of Italian physicians reported an estimated prevalence of hypertension and CVD between 10 and 20%. On the other hand, physicians reported a relatively high prevalence of cardiac organ damage, namely left ventricular hypertrophy, which has demonstrated high predictive value on the risk of hypertension-related CVD. This highlights the need for having specific diagnostic and therapeutic indications, in order to improve the clinical management of patients with hypertension and cardiac organ damage and reduce the potential risk of CVD.

In addition, Italian physicians considered the presence of cardiac organ damage, namely left ventricular hypertrophy, as the most common marker of organ damage. Also, the estimated prevalence of left ventricular hypertrophy was considered to be substantially higher than those reported for other hypertension-related markers of organ damage, including renal abnormalities and carotid atherosclerosis, thus highlighting the clinical relevance given by both groups of physicians to hypertension-related cardiac organ damage. However, the existence of left ventricular hypertrophy can be easily detected by simple ECG, whereas tests for other markers of organ damage except for cardiac one, may result in additional costs. Given this consideration, lower prevalence of some types of organ damage may be due to incomplete evaluation in a setting of real clinical practice.

Preferred options expressed by involved physicians for BP targets to be achieved in treated hypertensive patients with CVD resulted of particularly relevance, because of Italian GPs aimed to achieve more ambitious targets than those expressed by specialized physicians and recommended by current guidelines. In the most recent guidelines [12, 20], it has been stated that the therapeutic goals of antihypertensive treatment in patients with previous TIA or stroke were to reduce long-term risk of CVD complications and to achieve the recommended BP targets of 140/90 mmHg. In these hypertensive patients with CVD, all classes of antihypertensive drugs can be effectively used to reduce BP levels according to European guidelines [12], whereas those drugs able to inhibit the renin-angiotensin system, including ACE inhibitors and ARBs, and calcium-channel blockers should be preferred according to British guidelines [20], in order to reduce morbidity and mortality and improve event-free survival [21–24]. The main findings of the present survey are confident with these indications. In fact, among various pharmacological options, Italian physicians are clearly oriented for drugs inhibiting the renin-angiotensin system, both in monotherapy and in combination therapy. These drugs, including ACE inhibitors and ARBs, are considered by both groups of involved clinicians as the preferred drug options for treating hypertensive patients with CVD. In particular, GPs tended to prefer antihypertensive therapies based on ACE inhibitors, whereas specialised physicians expressed a clear preference for ARB-based therapies, both in monotherapies and in combination therapies with beta-blockers in all groups. Similar proportions have been also observed in previous analyses by the same education program [16, 17], thus confirming a general prescriptive trend by Italian physicians, which is substantially based on either ACE inhibitors in the setting of general practice or ARBs in the setting of specialised medicine. In this latter regard, it should be noted, however, that in Italy regulatory rules for antihypertensive drug prescriptions support a larger use of low-cost ACE inhibitors compared to that of ARBs, especially in the setting of general practice. Another potential explanation might be the fact that ACE inhibitors are mostly used as first-line strategy by GPs, whereas ARBs are predominantly used by SPs in hospital divisions and reference centers in those hypertensive patients at high or very high cardiovascular risk profile or in those in whom ACE inhibitors have lost their antihypertensive efficacy or caused side effects (mostly cough). Another aspect that should be noted is the preferred use of beta-blockers, both in monotherapy and mostly in combination therapies, expressed by involved physicians. This therapeutic choice seems to be not in line with recommendations from North American [25] and British [20] guidelines, although compelling indications from 2013 European guidelines stated that any antihypertensive drug class can be used for lowering BP levels in patients with hypertension and previous stroke or TIA, including beta-blockers [12].

### Potential limitations

As applied for previous analyses [16, 17], some potential limitations should be acknowledged. First of all, the present study is a descriptive survey, thus it can only describe physicians’ answers on how the manage hypertensive outpatients with CVD in their practice. Secondly, dependence on physician self-reporting, rather than more objective measures such as clinical records and pharmacological prescriptions, may be viewed as a potential bias. Inclusion of physicians from different geographical area, medical specialities and age of clinical activity may have at least, in part, affected the main findings of the present analysis, although all these aspects will be analysed in a predefined further analysis of the pooled data from derived from this educational program. The design of the study does not allow the evaluation of the treatment efficacy according to answers provided by involved physicians to questions about diagnostic tools, BP targets and preferred medications. Finally, the survey was largely driven by the answers reported by GPs, who predominantly participated to this study, rather than by those reported by cardiologists, neurologists or other professional figures, who may be involved in the clinical management of hypertensive outpatients with CVD.

## Conclusions

Although limited by the descriptive nature of the survey, this study provides some relevant information on attitudes and preferences, as well as on different diagnostic and therapeutic approaches applied by physicians when managing hypertensive outpatients with CVD in Italy. The main findings of our analysis, in fact, demonstrated that the concomitant presence of hypertension and cardiac markers of organ damage is reported to be high, whereas that of hypertension and CVD is considered to be relatively low in a setting of clinical practice by both groups of involved physicians. Even in the absence of specific indications from international guidelines, GPs tended to attain more ambitious BP targets in hypertensive outpatients with CVD. To achieve these BP targets, pharmacological therapies based on ACE inhibitors, either in monotherapy or combination therapy (mostly with beta-blockers), represented the preferred options.

## Additional file

Additional file 1: Table S1. Survey questionnaire. (DOCX 21 kb)

## Abbreviations

ACE: Angiotensin-converting enzyme
ARB: Angiotensin receptor blockers
BP: Blood pressure
CT: Computer tomography
CV: Cardiovascular
CVD: Cerebrovascular diseases
GPs: General practitioners
MR: Magnetic resonance
SPs: Specialized physicians
TIA: Transient ischemic attack
