# Supplementary material for: Attitudes and preferences for the clinical management of hypertension and hypertension-related cerebrovascular disease in the general practice: results of the Italian hypertension and brain survey
Source: Clin Hypertens. 2017 May 15;23:10. doi: 10.1186/s40885-017-0066-0 (PMC5430606; doi:10.1186/s40885-017-0066-0)
Supplement: Additional file 1: Table S1. — Survey questionnaire. (DOCX 21 kb) [file 40885_2017_66_MOESM1_ESM.docx]

**Additional file 1: Table S1 (online available).**

| **Question (num/text)** |
| --- |
|  |
| **Q01. Which is the most prevalent marker of organ damage do you find in patients with hypertension in your clinical practice?** |
| - Left Ventricular Hypertrophy |
| - Carotid Atherosclerosis |
| - Microalbuminuria or Proteinuria |
| - Impaired eGFR or CrCl |
| - Impaired ABI or PWV |
|  |
| **Q02. Which is the prevalence of cardiac organ damage (i.e. left ventricular hypertrophy) do you find in patients with hypertension in your clinical practice?** |
| - 10-20% |
| - 21-40% |
| - 41-50% |
| - >50% |
|  |
| **Q03. Which is the prevalence of renal organ damage (i.e. MAU, proteinuria, reduced eGFR or creatinine clearance) do you find in patients with hypertension in your clinical practice?** |
| - 10-20% |
| - 21-40% |
| - 41-50% |
| - >50% |
|  |
| **Q04. Which is the prevalence of vascular organ damage (i.e. carotid or peripheral atherosclerosis) do you find in patients with hypertension in your clinical practice?** |
| - 10-20% |
| - 21-40% |
| - 41-50% |
| - >50% |
|  |
| **Q05. Which is the prevalence of cerebrovascular disease (i.e. transient ischemic attack) do you find in patients with hypertension in your clinical practice?** |
| - 10-20% |
| - 21-40% |
| - 41-50% |
| - >50% |
|  |
| **Q06. Which is the prevalence of cerebrovascular disease (i.e. stroke) do you find in patients with hypertension in your clinical practice?** |
| - 10-20% |
| - 21-40% |
| - 41-50% |
| - >50% |
|  |
| **Q07. Which diagnostic tool do you think is the most appropriate in patients with hypertension and CVD (i.e. transient ischemic attack or stroke) in your clinical practice?** |
| - Echocardiogram |
| - Carotid Vascular Ultrasound |
| - Transcranic Vascular Ultrasound |
| - 24-hour ABPM |
| - Central Aortic Pressure and/or PWV |

|  |
| --- |
| **Q08. Which diagnostic tool do you think is the most appropriate in patients with hypertension to exclude the presence of CVD (i.e. transient ischemic attack or stroke) in your clinical practice?** |
| - Carotid Vascular Ultrasound |
| - Transcranic Vascular Ultrasound |
| - Electroencefalogram |
| - Brain Imaging (CT or MR) |
| - Angio-MR |
|  |
| **Q09.** **Which is the most appropriate blood pressure target to be in patients** **with hypertension and transient ischemic attack in your clinical practice?** |
| - <150/90 mmHg |
| - <140/90 mmHg |
| - <135/85 mmHg |
| - <130/80 mmHg |
| - <120/80 mmHg |
|  |
| **Q10. Which is the most important target do you wish to achieve in patients with hypertension and transient ischemic attack in your clinical practice?** |
| - Reduce BP levels |
| - Achieve the recommended BP targets |
| - Protect from organ damage |
| - Improve adherence and persistence on therapy |
| - Reduce side effects and adverse reactions |
|  |
| **Q11. Which is the first line therapy to do you wish to use in patients** **with hypertension and transient ischemic attack in monotherapy in your clinical practice?** |
| - ACE inhibitors |
| - Angiotensin receptor blockers |
| - Beta-Blockers |
| - Calcium-channel blockers |
| - Diuretics |
|  |
| **Q12. Which is the combination therapy to do you wish to use in patients** **with hypertension and transient ischemic attack in your clinical practice?** |
| - ACE inhibitors + Diuretics |
| - ACE inhibitors + Calcium-channel blockers |
| - ACE inhibitors + Beta-Blockers |
| - Angiotensin receptor blockers + Diuretics |
| - Angiotensin receptor blockers + Calcium-channel blockers |
| - Angiotensin receptor blockers + Beta-Blockers |
| - Beta-Blockers + Diuretics |
| - Direct Renin Inhibitors + Diuretics |
|  |
| **Q13.** **Which is the most appropriate blood pressure target to be in patients** **with hypertension and previous stroke in your clinical practice?** |
| - <150/90 mmHg |
| - <140/90 mmHg |
| - <135/85 mmHg |
| - <130/80 mmHg |
| - <120/80 mmHg |
|  |

| **Q14. Which is the most important target do you wish to achieve in patients with hypertension and previous stroke in your clinical practice?** |
| --- |
| - Reduce BP levels |
| - Achieve the recommended BP targets |
| - Protect from organ damage |
| - Improve adherence and persistence on therapy |
| - Reduce side effects and adverse reactions |
|  |
| **Q15. Which is the first line therapy to do you wish to use in patients** **with hypertension and previous stroke in monotherapy in your clinical practice?** |
| - ACE inhibitors |
| - Angiotensin receptor blockers |
| - Beta-Blockers |
| - Calcium-channel blockers |
| - Diuretics |
|  |
| **Q16. Which is the combination therapy to do you wish to use in patients** **with hypertension and previous in your clinical practice?** |
| - ACE inhibitors + Diuretics |
| - ACE inhibitors + Calcium-channel blockers |
| - ACE inhibitors + Beta-Blockers |
| - Angiotensin receptor blockers + Diuretics |
| - Angiotensin receptor blockers + Calcium-channel blockers |
| - Angiotensin receptor blockers + Beta-Blockers |
| - Beta-Blockers + Diuretics |
| - Direct Renin Inhibitors + Diuretics |
|  |
